# Diagnostic Value of Biochemical Markers in the Preoperative and Intraoperative Verification of Biliary Fistulas in Liver Echinococcosis

**DOI:** 10.7759/cureus.81951

**Published:** 2025-04-09

**Authors:** Azam Babadjanov, Akbar Bazarbaev, Gayratbek Saliev

**Affiliations:** 1 Department of Hepatobiliary Surgery and Liver Transplantation No. 2, Republican Specialized Scientific and Practical Medical Center of Surgery Named After Academician V. Vakhidov, Tashkent, UZB; 2 Department of Surgery, Andijan State Medical Institute, Andijan, UZB

**Keywords:** biliary fistulas, cystic echinococcosis, hydatid cyst, liver echinococcosis, occult and obvious biliary fistulas

## Abstract

Objective

This study evaluates the prognostic significance of biochemical markers in diagnosing biliary fistulas (BF) in liver echinococcosis (LE).

Methods

The study included 85 patients with LE who underwent surgery between 2021 and 2024. Among them, 24 had BF (11 men, 13 women, mean age 40.5±14), and 61 had no BF (27 men, 34 women, mean age 38±13.4). Cysts were classified by WHO-IWGE criteria. Biochemical markers (total and conjugated bilirubin, ALP, GGT, ALT, AST, leukocyte count, eosinophils) were analyzed in blood and cyst contents. Statistical analysis used independent t-tests (p<0.05).

Results

Preoperative blood analysis showed no significant differences in bilirubin, ALP, GGT, ALT, AST, or leukocyte count between groups (p>0.05). However, eosinophil levels were significantly higher in the BF group (5.8±3.8% vs. 3.7±2.9%, p=0.0184).

Cyst content analysis revealed significantly higher total bilirubin in BF cysts (28.8±21.4 µmol/L) versus non-BF cysts (1.2±0.9 µmol/L, p=0.00002). Conjugated bilirubin was also higher (16±15.3 µmol/L vs. 0.1±0.3 µmol/L, p=0.00018), while ALP and GGT showed no significant differences (p>0.05).

Subgroup analysis (CE1-2 vs. CE3) showed no significant differences in biochemical markers between BF and non-BF groups (p>0.05), except eosinophils, which were higher in the BF group (p=0.0080). In CE1-2, occult BF cysts had higher total bilirubin than non-BF cysts (6±3.7 µmol/L vs. 1.2±1 µmol/L, p=0.0132). In CE3, total bilirubin was also significantly higher in BF cysts (p=0.00002).

Further analysis showed that total bilirubin in obvious BF was 41±15.9 µmol/L, significantly higher than in occult BF (6±3.7 µmol/L, p<0.00001).

Conclusion

Biochemical analysis of cyst contents is superior to blood markers for BF verification. Total bilirubin in cyst contents had 100% specificity and 92.0% accuracy, with sensitivity increasing from 60.0% to 95% at a threshold of 2.6 µmol/L. Blood markers, including bilirubin and GGT, had limited diagnostic value. Elevated eosinophils (p=0.0184) were the only significant blood marker but lacked sufficient sensitivity (58.3%) and specificity (72.1%) for reliable preoperative diagnosis.

## Introduction

Cystic echinococcosis (CE) is one of the most common zoonotic diseases and an important public health problem in regions such as the Mediterranean, North Africa, Southern and Eastern Europe, areas of South America, Central Asia, Siberia, and Western China [1]. Liver echinococcosis (LE) ranks first in frequency of occurrence among other organs [2-4]. In modern surgery of LE, biliary fistulas (BF) are the most common complication [5], with frequency varying from 2.6 to 28.6% [6] depending on various factors, and according to some sources - from 13 to 37% [7,8].

Two types of BF pathogenesis are known: the first involves compression of the bile duct wall by the hepatic echinococcal cyst (EC), leading to BF formation; the second mechanism suggests that after capturing small bile ducts in the pericystic wall, high intracystic pressure first causes their atrophy and then leads to rupture of bile ducts into the cyst [9]. Although most BFs close spontaneously, in some cases, bile leakage leads to a complicated postoperative course, and wait-and-see tactics are not always justified, especially in cases where bile output exceeds 100 ml [10].

The literature also describes two variants of BF: obvious and occult [11]. An obvious BF is an open connection between the cyst cavity and intrahepatic bile ducts, which allows cyst contents to drain directly into the bile duct and also promotes bile penetration into the cyst cavity [12]. Obvious BFs are easier to diagnose, while occult BFs do not show clear symptoms, and their diagnosis is significantly difficult, even directly during surgery [13]. Results of some studies have shown a statistically significant relationship between biochemical parameters of cystic fluid and the development of biliary complications, as well as the influence of formed cysto-biliary fistulas on increasing LE recurrence frequency. In particular, assessment of alkaline phosphatase (ALP) and gamma-glutamyltransferase (GGT) values can predict the presence of BF in hepatic echinococcal cysts [14]. Another study also recommends the GGT indicator for predicting occult cysto-biliary fistula [15].

However, despite existing publications on studies of changes in clinical and biochemical parameters in the aspect of verifying BF development in LE, there is still no consensus on their effectiveness, as some literature sources may confirm the clinical significance of specific markers, while others refute it. Therefore, we conducted our analysis of laboratory data of patients with BF in LE in comparison with a group of patients with LE without this complication.

The study aims to evaluate the prognostic significance of biochemical markers in the diagnosis of biliary fistulas in liver echinococcosis.

## Materials and methods

This study is registered with ClinicalTrials.gov (ID NCT06612229). The study included 85 patients with LE operated on in 2021-2024, with 24 patients in the BF group, of whom 11 (45.8%) were men and 13 (54.2%) were women, mean age of 40.5±14, and in the group without BF, 61 patients including 27 (44.3%) men and 34 (55.7%) women, mean age of this group was 38±13.4. Cysts were also divided according to WHO (WHO-IWGE) classification [16], which is based on ultrasound characteristics of CE 1-4 cyst development.

The study included patients who were over 18 years of age and had a confirmed diagnosis of liver echinococcosis based on instrumental diagnostic methods. All participants underwent conservative surgical treatment and had a complete preoperative examination. Additionally, a detailed description of the surgical intervention was required, along with comprehensive data on the postoperative period. Patients were excluded from the study if they were under 18 years of age or had extrahepatic echinococcosis. Those with incomplete diagnostic data or severe comorbidities that could significantly impact the study outcomes were also not included. All patients underwent a thorough evaluation, which encompassed general clinical assessment, laboratory tests, and instrumental diagnostic methods.

Clinical and biochemical studies were conducted to assess both the cyst contents and blood parameters of the patients. The biochemical analysis of the cyst contents included measurements of total bilirubin, conjugated bilirubin, alkaline phosphatase (ALP), and gamma-glutamyl transferase (GGT). Additionally, blood analysis was performed to evaluate total and conjugated bilirubin, ALP, GGT, alanine aminotransferase (ALT), and aspartate aminotransferase (AST). The blood tests also included an assessment of leukocyte count and the percentage of eosinophils, providing a comprehensive understanding of the patients' biochemical and hematological status.

Biochemical parameters were determined using a Vitros 5600 (Ortho Clinical Diagnostic, Raritan, NJ, USA) automatic analyzer, and hematological parameters - on a DxH-500 analyzer (Beckman-Coulter, Brea, CA, USA).

To assess the diagnostic significance of various criteria, sensitivity, specificity, and accuracy indicators were calculated. Categorical variables are presented as absolute numbers and percentages. Quantitative data are presented as mean ± standard deviation (M±δ) with a 95% confidence interval (CI), independent t-test. Differences were considered statistically significant at p<0.05.

## Results

**Figure 1:**
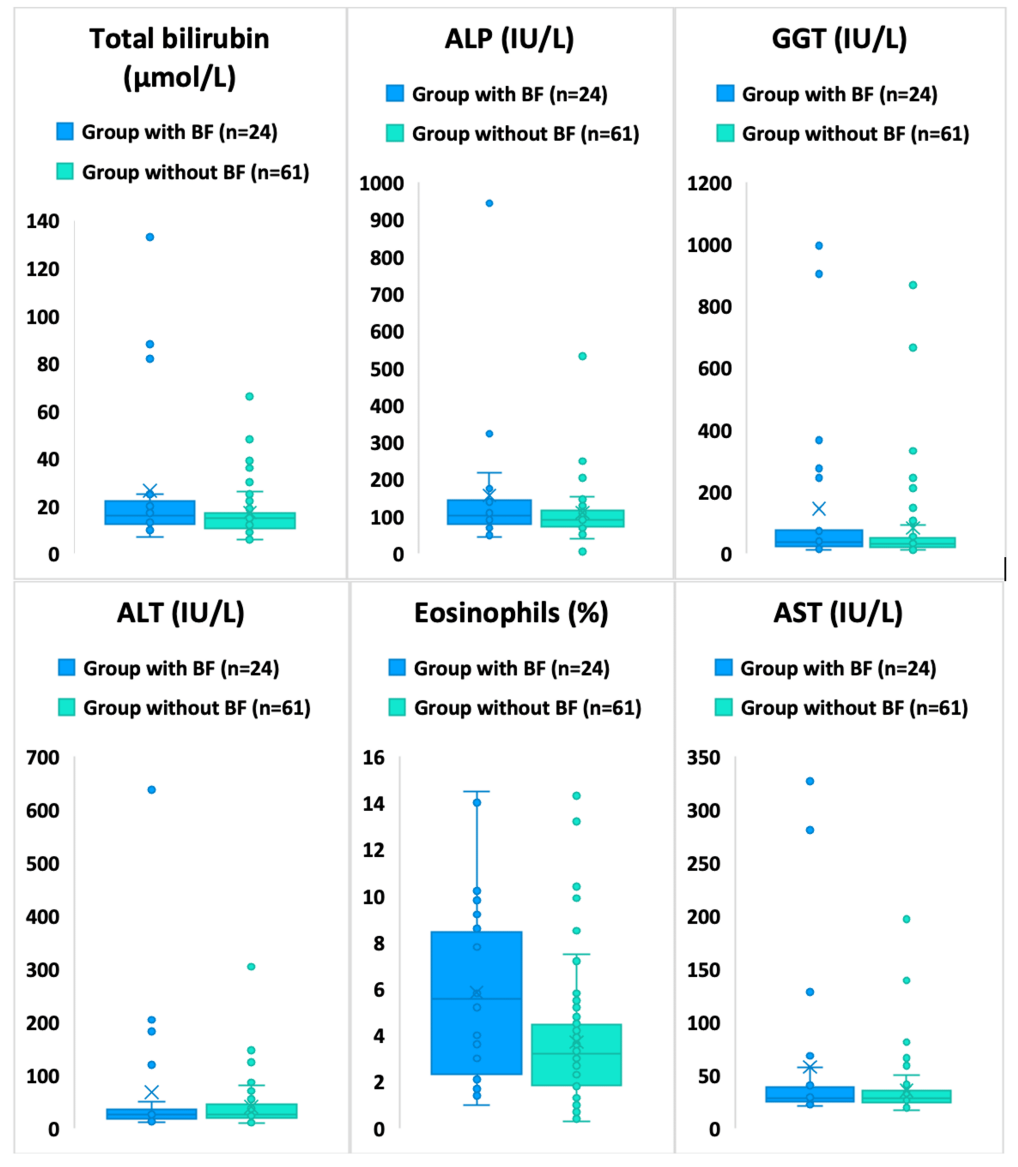
Some clinical and biochemical blood parameters overall in groups with and without BF. BF: biliary fistulas; ALP: alkaline phosphatase; GGT: gamma-glutamyltransferase; ALT: alanine aminotransferase; AST: aspartate aminotransferase.

Biochemical blood analysis of patients with LE and BF and a comparison group with LE without BF at the preoperative level has been completed, and all relevant laboratory data are provided in the manuscript. Results of total and conjugated bilirubin, ALP, GGT, ALT, AST, blood leukocytes (x10^9^/L), and eosinophils (%) were studied. In some publications regarding BF verification in EC, authors cite sensitivity of cholestasis markers (total bilirubin, ALP, GGT indicators) and eosinophils. Our studies showed no statistically significant differences in preoperative levels of cholestasis markers both overall between groups (with BF and without BF) and by parasite development stages. This part of the study was conducted in 24 patients with LE with BF and 61 patients with LE without BF. Thus, when comparing data in patients with and without BF, total bilirubin indicators were 26.5±30.3 μmol/L and 17.2±12.5 μmol/L respectively, conjugated bilirubin 4.3±13.4 versus 0.5±2.1 μmol/L (p>0.05). ALP, GGT, ALT, and AST indicators also showed no differences between groups (Figure 1 and Table 1). Both groups had patients with elevated levels of these markers.

**Table 1:** Some clinical and biochemical blood parameters overall in groups with and without BF. BF: biliary fistulas; ALP: alkaline phosphatase; GGT: gamma-glutamyltransferase; ALT: alanine aminotransferase; AST: aspartate aminotransferase.

Parameter	Group with BF (n=24)				Group without BF (n=61)				t-test	
	Min	Max	M±δ	95%CI	Min	Max	M±δ	95%CI	value	p
Total bilirubin (μmol/L)	7.0	133.0	26.5±30.3	13.7-39.2	6.0	68.0	17.2±12.5	14-20.4	1.45	0.160
Conjugated bilirubin (μmol/L)	0.0	59.0	4.3±13.4	-1.3-10	0.0	12.0	0.5±2.1	0-1	1.40	0.176
ALP (IU/L)	44.0	944.0	157±183.3	79.6-234.4	6.0	548.0	110.4±87.9	87.9-132.9	1.19	0.243
GGT (IU/L)	11.0	996.0	144.5±264.4	32.8-256.1	11.0	867.0	82.4±148.3	44.4-120.4	1.08	0.287
ALT (IU/L)	11.0	637.0	67.8±131.5	12.2-123.3	9.0	304.0	40.3±45	28.8-51.9	1.00	0.328
AST (IU/L)	21.0	327.0	57.6±79.4	24.1-91.2	17.0	197.0	35.4±27.6	28.3-42.5	1.34	0.193
Leukocytes (10^9^/L)	3.3	15.6	6.8±2.6	5.7-7.9	2.4	20.0	6.9±3	6.1-7.7	-0.15	0.882
Eosinophils (%)	1.0	14.5	5.8±3.8	4.2-7.4	0.3	14.3	3.7±2.9	3-4.4	2.48	0.018

When considering fluctuations in cholestasis markers, it is evident that their elevation cannot serve as a reliable risk factor for BF development due to the lack of statistical significance. In the group without BF, there were patients with large cysts that compressed bile ducts, causing the development of cholestasis syndrome. Elevation of cytolysis markers is also not specific to BF, as various factors can cause it. The only sensitive marker was the eosinophil level, which was significantly higher in the BF group (5.8±3.8% versus 3.7±2.9%; p=0.0184) (Figure 1). This is associated with the contact between cyst contents and the bile duct, which leads to the development of a response reaction.

It turned out that even if we consider only EC in stage CE1-2 (42 patients) in the group without BF, excluding CE3 from the sample, no significant differences in cholestasis and cytolysis markers were detected, confirming the lack of sensitivity of these indicators to attempts at verifying BF in EC at the preoperative stage. The exception was again the blood eosinophil indicator (Table 2).

**Table 2:** Some clinical and biochemical parameters in LE with BF and LE without BF in stage CE1-2 LE: liver echinococcosis; BF: biliary fistulas; ALP: alkaline phosphatase; GGT: gamma-glutamyltransferase; ALT: alanine aminotransferase; AST: aspartate aminotransferase.

Parameter	Group with BF (n=24)				Group without BF (n=61)				t-test	
	Min	Max	M±δ	95%CI	Min	Max	M±δ	95%CI	value	p
Total bilirubin (μmol/L)	7.0	133.0	26.5±30.3	13.7-39.2	6.0	66.0	16.8±11.7	13.2-20.5	1.49	0.147
Conjugated bilirubin (μmol/L)	0.0	59.0	4.3±13.4	-1.3-10	0.0	10.0	0.4±1.8	-0.1-1	1.42	0.170
ALP (IU/L)	44.0	944.0	157±183.3	79.6-234.4	6.0	548.0	108.5±81.4	83.2-133.9	1.23	0.230
GGT (IU/L)	11.0	996.0	144.5±264.4	32.8-256.1	11.0	867.0	82.4±145.4	37.1-127.7	1.06	0.297
ALT (IU/L)	11.0	637.0	67.8±131.5	12.2-123.3	9.0	304.0	38.9±48.6	23.7-54	1.04	0.309
AST (IU/L)	21.0	327.0	57.6±79.4	24.1-91.2	17.0	197.0	33.6±27.2	25.1-42.1	1.43	0.163
Leukocytes (10^9^/L)	3.3	15.6	6.8±2.6	5.7-7.9	3.3	20.0	7.3±3.3	6.2-8.3	-0.61	0.543
Eosinophils (%)	1.0	14.5	5.8±3.8	4.2-7.4	0.3	14.3	3.3±2.9	2.4-4.2	2.80	0.0080

In turn, a comparison of equivalent subgroups with EC development stage CE1-2 showed that in the BF group, total bilirubin was 22±24.9 μmol/L (Table 3), and in the group without BF - 16.8±11.7 μmol/L (p>0.05) (Table 2). Other parameters also showed no significant differences (Table 3).

**Table 3:** Some clinical and biochemical parameters in LE at stage CE1-2 with BF Note: Data for the group without BF with stage CE1-2 are presented in Table 2. LE: liver echinococcosis; BF: biliary fistulas; ALP: alkaline phosphatase; GGT: gamma-glutamyltransferase; ALT: alanine aminotransferase; AST: aspartate aminotransferase.

Parameter	Group with BF (n=9)				t-test	
	Min	Max	M±δ	95%CI	value	p
Total bilirubin (μmol/L)	9	11.0	88.0	22±24.9	0.61	0.558
Conjugated bilirubin (μmol/L)	9	0.0	29.0	3.2±9.7	0.86	0.414
ALP (IU/L)	9	44.0	944.0	193.9±286.4	0.89	0.400
GGT (IU/L)	9	14.0	903.0	158.3±290.9	0.76	0.466
ALT (IU/L)	9	11.0	204.0	60.1±76.4	0.80	0.443
AST (IU/L)	9	21.0	281.0	60.4±83.9	0.95	0.369
Leukocytes (10^9^/L)	9	3.3	15.6	6.3±3.8	-0.74	0.478
Eosinophils (%)	9	1.0	10.2	5.5±3.2	1.86	0.090

Similar data were obtained in subgroups with CE3 stage, where total bilirubin in the BF group was 29.1±33.7 μmol/L, and in the group without BF - 18.1±14.4 μmol/L (p>0.05). Also, no significant differences were found in levels of ALT, AST, and other parameters. Eosinophils had somewhat higher values in the BF group (6±4.2% versus 4.5±2.8%), but the difference did not reach statistical significance (p=0.2428) (Table 4).

**Table 4:** Some clinical and biochemical parameters in LE with BF and LE without BF at stage CE3 LE: liver echinococcosis; BF: biliary fistulas; ALP: alkaline phosphatase; GGT: gamma-glutamyltransferase; ALT: alanine aminotransferase; AST: aspartate aminotransferase.

Parameter	Group with BF (n=15)				Group without BF (n=19)				t-test	
	Min	Max	M±δ	95%CI	Min	Max	M±δ	95%CI	value	p
Total bilirubin (μmol/L)	7.0	133.0	29.1±33.7	10.5-47.8	7.0	68.0	18.1±14.4	11.2-25	1.19	0.2509
Conjugated bilirubin (μmol/L)	0.0	59.0	5±15.5	-3.6-13.6	0.0	12.0	0.6±2.8	-0.7-2	1.08	0.2982
ALP (IU/L)	71.0	334.0	134.9±83.3	88.7-181	63.0	531.0	114.4±103.2	64.7-164.2	0.64	0.5276
GGT (IU/L)	11.0	996.0	136.1±257.5	-6.4-278.7	11.0	666.0	82.3±158.8	5.8-158.8	0.71	0.4850
ALT (IU/L)	14.0	637.0	72.3±158.2	-15.3-159.9	11.0	136.0	43.6±36.9	25.8-61.3	0.69	0.5010
AST (IU/L)	22.0	327.0	55.9±79.6	11.8-100	19.0	139.0	39.4±28.9	25.4-53.3	0.77	0.4539
Leukocytes (10^9^/L)	4.2	9.9	7.1±1.6	6.3-8	2.4	10.1	6.1±2.2	5.1-7.2	1.55	0.1315
Eosinophils (%)	1.4	14.5	6±4.2	3.7-8.4	0.7	13.2	4.5±2.8	3.2-5.9	1.20	0.2428

**Figure 2:**
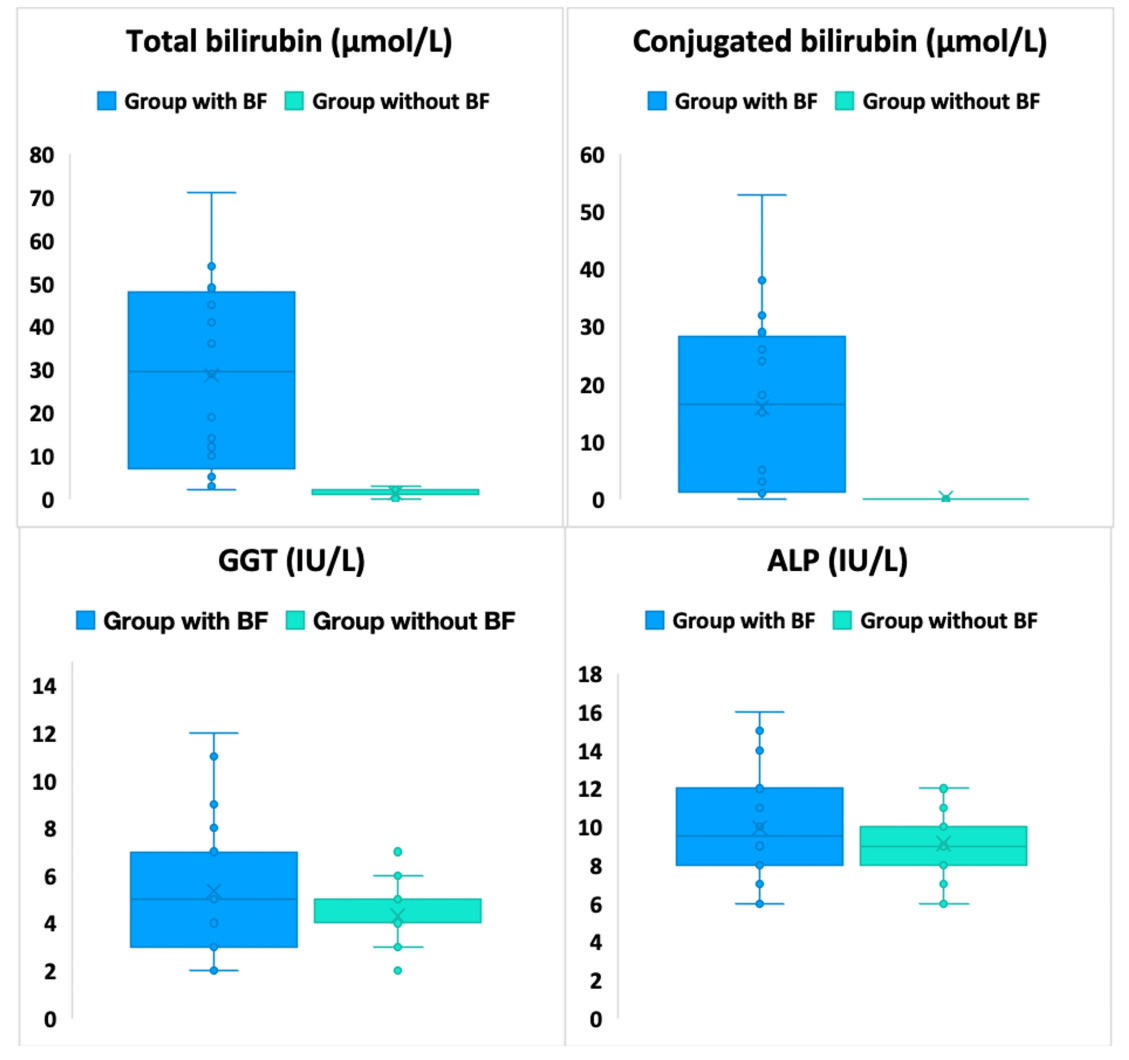
Some biochemical parameters of cyst contents overall in groups with and without BF BF: biliary fistulas; ALP: alkaline phosphatase; GGT: gamma-glutamyltransferase

Comparative analysis of biochemical parameters of EC contents in the liver revealed substantial differences between groups with BF (20 cysts) and without BF (80 cysts). Total bilirubin levels in cysts with BF were significantly higher (28.8±21.4 μmol/L) compared to groups without BF (1.2±0.9 μmol/L, p=0.00002). Conjugated bilirubin was also significantly higher in the BF group (16±15.3 μmol/L) compared to the group without BF (0.1±0.3 μmol/L, p=0.00018). ALP and GGT parameters showed no significant differences between groups, indicating a lack of sensitivity of this test to the presence of BF in cysts (Figure 2 and Table 5).

**Table 5:** Some biochemical parameters of cyst contents overall in groups with and without BF BF: biliary fistulas; ALP: alkaline phosphatase; GGT: gamma-glutamyltransferase

Parameter	Group with BF					Group without BF					t-test	
	n	Min	Max	M±δ	95%CI	n	Min	Max	M±δ	95%CI	value	p
Total bilirubin (μmol/L)	20	2	71	28.8±21.4	18.7-40.3	80	0	3	1.2±0.9	1-1.4	5.75	0.00002
Conjugated bilirubin (μmol/L)	20	0.0	53	16±15.3	8.8-24.2	80	0	1	0.1±0.3	0-0.2	4.63	0.00018
ALP (IU/L)	20	6.0	16	10±3.2	8.5-11.7	80	6	12	9.1±1.7	8.7-9.5	1.13	0.27153
GGT (IU/L)	20	2.0	12	5.4±2.9	4-6.9	80	2	7	4.3±1.3	4-4.6	1.56	0.13377

The next stage involved a separate analysis of the biliary fistula (BF) group with division into obvious and occult BF, which showed that obvious BF was associated with higher levels of total and conjugated bilirubin. Total bilirubin in the obvious BF group was 41±15.9 μmol/L, while in the occult BF group it was significantly lower (6±3.7 μmol/L, p<0.00001). The level of total bilirubin in the blood of patients with BF was 26.5±30.3 μmol/L, in the group without BF - 17.2±12.5 μmol/L, however, the differences did not reach statistical significance (p>0.05). A similar situation was observed for conjugated bilirubin: 4.3±13.4 μmol/L in the BF group versus 0.5±2.1 μmol/L in the group without BF (p>0.05) (Figure 1).

Comparison between cysts with BF and cysts without BF in the CE3 stage also demonstrated a significant increase in bilirubin in the BF group. Total bilirubin in the BF group was significantly higher compared to the group with EC in the transitional stage without BF (1.4±0.7 μmol/L, p=0.00002). Conjugated bilirubin in this group was also substantially lower (0±0.2 μmol/L, p=0.00017) (Table 6).

**Table 6:** Some biochemical parameters of cyst contents in patients with BF and without BF in the CE3 stage. BF: biliary fistulas; ALP: alkaline phosphatase; GGT: gamma-glutamyltransferase

Parameter	Group with BF					Group without BF					t-test	
	n	Min	Max	M±δ	95%CI	n	Min	Max	M±δ	95%CI	value	p
Total bilirubin (μmol/L)	20	2	71	28.8±21.4	18.7-40.3	21	0	3	1.4±0.7	1.1-1.7	5.71	0.00002
Conjugated bilirubin (μmol/L)	20	0.0	53	16±15.3	8.8-24.2	21	0	1	0±0.2	-0.1-0.1	4.65	0.00017
ALP (IU/L)	20	6.0	16	10±3.2	8.5-11.7	21	6	12	8.8±2	7.9-9.7	1.44	0.16022
GGT (IU/L)	20	2.0	12	5.4±2.9	4-6.9	21	2	7	4±1.1	3.5-4.6	1.86	0.07571

The level of conjugated bilirubin was also higher in the obvious BF group (23.8±13.3 μmol/L versus 1.3±1 μmol/L, p=0.00005) (Table 7). At the same time, both parameters in the occult BF group were also significantly higher than in the group without LE fistulas (Table 8). Analysis of blood biochemical parameters showed less pronounced differences between groups.

**Table 7:** Some biochemical parameters of cyst contents in patients with occult BF and without BF BF: biliary fistulas; ALP: alkaline phosphatase; GGT: gamma-glutamyltransferase

Parameter	Obvious BF					Occult BF					t-test	
	n	Min	Max	M±δ	95%CI	n	Min	Max	M±δ	95%CI	value	p
Total bilirubin (μmol/L)	13	14	71	41±15.9	31.4-50	7	2	12	6±3.7	2.6-9.4	7.56	<0.0001
Conjugated bilirubin (μmol/L)	13	3.0	53	23.8±13.3	15.8-31.3	7	0	3	1.3±1	0.4-2.2	6.09	0.00005
ALP (IU/L)	13	6.0	16	10.5±3.6	8.3-12.6	7	6	11	8.9±1.7	7.3-10.4	1.41	0.17581
GGT (IU/L)	13	2.0	12	6±3.3	4-7.9	7	2	7	4.1±1.7	2.6-5.7	1.66	0.11369

**Table 8:** Some biochemical parameters of cyst contents in patients with occult BF and without BF in CE1-2 stages BF: biliary fistulas; ALP: alkaline phosphatase; GGT: gamma-glutamyltransferase

Parameter	Group with BF					Group without BF					t-test	
	n	Min	Max	M±δ	95%CI	n	Min	Max	M±δ	95%CI	value	p
Total bilirubin (μmol/L)	7	2.0	12.0	6±3.7	2.6-8.3	59	0.0	3.0	1.2±1	0.9-1.4	3.45	0.0132
Conjugated bilirubin (μmol/L)	7	0.0	3.0	1.3±1	0.4-1.9	59	0.0	1.0	0.1±0.3	0-0.2	3.22	0.0174
ALP (IU/L)	7	6.0	11.0	8.9±1.7	7.3-9.9	59	6.0	12.0	9.3±1.6	8.8-9.7	-0.59	0.5703
GGT (IU/L)	7	2.0	7.0	4.1±1.7	2.6-5.2	59	2.0	7.0	4.4±1.3	4.1-4.7	-0.38	0.7178

Analysis of groups with obvious and occult BF showed that obvious BF was associated with higher levels of total and conjugated bilirubin. Total bilirubin in the obvious BF group was 41±15.9 μmol/L, while in the occult BF group it was significantly lower (6±3.7 μmol/L, p<0.00001). The level of conjugated bilirubin was also higher in the obvious BF group (23.8±13.3 μmol/L versus 1.3±1 μmol/L, p=0.00005) (Table 7). At the same time, both parameters in the occult BF group were also significantly higher than in the group without fistulas with CE1-2 stages of LE (Table 8).

Thus, the study results showed that in terms of analyzing EC contents performed intraoperatively, only total and conjugated bilirubin levels proved to be maximally informative for confirming the presence of BF. While verification of obvious fistulas after cyst opening presents no difficulties due to bile imbibition (total bilirubin level - 41±15.9 μmol/L), in the presence of occult fistulas, the bilirubin level (6.0±3.7 μmol/L) (Table 7) was also significantly higher than in cysts without fistulas in CE1-2 (1.2±1 μmol/L; t=3.45; p=0.0132) (Table 8) and CE3 stages (1.4±0.7 μmol/L; t=3.25; p=0.0168) (Table 6).

These data allowed supplementing information about BF differentiation. Obvious BF is verified pre-operatively as cyst content breakthrough into the biliary tract, as well as complete or partial bile imbibition of cyst contents. The bilirubin level in cyst contents in these cases averaged 41.0±15.9 μmol/L (95% CI 31.4-50.0 μmol/L). For occult BF, pre-operative diagnostic effectiveness is less sensitive, while rapid biochemical analysis of cyst contents showed an average total bilirubin level of 6.0±3.7 μmol/L (95% CI - 2.6-8.3 μmol/L) (Table 7). It should be noted that in doubtful cases during rapid analysis of cyst contents, detection of total bilirubin above 2.6 μmol/L will indicate the presence of an occult BF.

From the obtained data, sensitivity, specificity, and accuracy indicators were calculated for various diagnostic criteria in LE with BF presence. Criteria having mean values (M±δ) were determined from the overall sample results, where the verified initial constant for differentiation of EC with and without BF was taken as the minimum value from the confidence interval (CI). A fundamental point is that all considered diagnostic criteria had low or medium sensitivity, indicating their potentially low diagnostic significance specifically for verifying the presence of BF.

**Figure 3:**
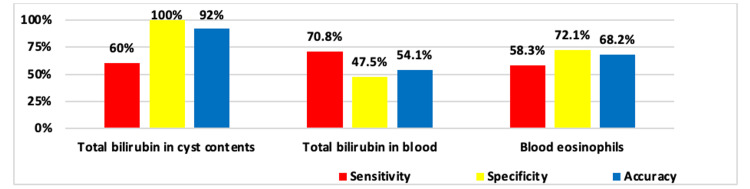
Determination of sensitivity, specificity, and accuracy of various criteria in the differential diagnosis of EC with BF

The study results showed that the total bilirubin level in cyst contents is the most reliable diagnostic criterion. This indicator has absolute specificity (100%) and high accuracy (92.0%), meaning that elevated bilirubin levels in cyst contents definitively indicate the presence of BF. The sensitivity of this criterion is 60.0% (Figure 3), which is also acceptable for clinical practice, and the low value of this indicator is associated with the presence of occult forms of the disease in the BF group. Accordingly, if the average bilirubin level in the BF group was 28.8±21.4 μmol/L with 95% CI 18.7-40.3 μmol/L (Table 2), and the sensitivity of this criterion was determined by this value, then in the occult BF group this indicator was 6.0±3.7 μmol/L (95% CI 2.6-8.3 μmol/L) versus 1.2±1.0 μmol/L (95% CI 0.9-1.4 μmol/L) in the group without BF, which was also significantly higher (p<0.001) (Table 8). If the sensitivity calculation is based on the level of 2.6 μmol/L, then the sensitivity degree increases to 95%.

However, some criteria showed limited diagnostic value due to low specificity. For example, blood total bilirubin levels had high sensitivity (83.3% and 70.8% respectively) but low specificity (47.4% and 47.5%) (Figure 3). This can lead to a large number of false-positive results, which limits the use of this method as the primary diagnostic tool for BF (Table 9).

**Table 9:** Determination of sensitivity, specificity, and accuracy of various criteria in the differential diagnosis of EC with BF Note: TP - True Positive; FP - False Positive; TN - True Negative; FN - False Negative cases; Se - Sensitivity; Sp - Specificity; Ac - Accuracy

Parameter	ТР	FP	TN	FN
Total bilirubin in cyst contents	12	0	80	8
Total bilirubin in blood	17	32	29	7
Blood eosinophils	14	17	44	10

The results of this phase of the study showed that in terms of EC contents analysis performed intraoperatively, only total and conjugated bilirubin levels proved maximally informative for confirming BF presence. While verification of obvious fistulas after cyst opening presents no difficulties due to bile imbibition (total bilirubin level - 41±15.9 μmol/L), in the presence of occult fistulas, the bilirubin level (6.0±3.7 μmol/L) (Table 7) was also significantly higher than in cysts without fistulas in CE1-2 (1.2±1 μmol/L; t=3.45; p=0.0132) (Table 8) and CE3 stages (1.4±0.7 μmol/L; t=3.25; p=0.0168) (Table 6).

In turn, clinical and biochemical blood parameters, despite some differences, have limited sensitivity and specificity for reliable preoperative detection of BF in LE. The only sensitive marker was the eosinophil level, which was significantly higher in the BF group (5.8±3.8% versus 3.7±2.9%; p=0.0184) (Figure 1), which is associated with cyst contents contact with the bile duct, causing a response reaction.

## Discussion

This study conducted a comprehensive analysis of biochemical parameters as predictors of BF development in LE. Analysis of cyst contents revealed significant differences in bilirubin levels between groups with and without BF, with differences between obvious and occult fistulas being particularly demonstrative. Among blood biochemical parameters, only eosinophil levels showed statistically significant differences between groups, while other markers of cholestasis and cytolysis showed no significant differences.

Our results largely differ from those of Demircan et al. [17] and Unalp et al. [13]. Their study noted an increased risk of BF development with elevated blood levels of AST (59.7 U/L), ALT (57.2 U/L), ALP (481.7 U/L), total bilirubin (33.9 µmol/L), conjugated bilirubin (21.4 µmol/L), and eosinophil count compared to groups without BF. Our data did not reveal significant differences regarding these parameters, except for eosinophil count. Studies by Alan et al. [18] also noted the significance of elevated ALP (235.04 U/L) and GGT (215.06 U/L), total bilirubin (1.51 mg/dL), conjugated bilirubin (0.96 mg/dL) in blood as BF predictors. Examination of these parameters in our presented patient cohort did not confirm these data, as all indicated markers did not significantly differ between patients with and without BF. Meanwhile, our cyst fluid analysis results align with Habeeb et al.'s study, which found high statistical significance in the difference of total and conjugated bilirubin levels inside cysts between groups with and without BF [16].

It should be noted that our study has several limitations. First, it's the retrospective nature of the analysis which may affect data accuracy. Second, the study was conducted in a single center, which may limit the result generalizability. Third, long-term patient follow-up was not conducted, preventing the assessment of long-term results and possible late complications. Additionally, not all patients had a complete set of biochemical parameters, which could have affected the statistical error of the study.

Despite these limitations, the obtained results have important practical significance. They allow for identifying the most reliable predictors of BF presence in liver echinococcosis - cyst size and bilirubin levels in their contents. These parameters can be used for preoperative assessment of biliary complications risk and planning appropriate surgical tactics. Further prospective multicenter studies are needed to validate the obtained results and develop standardized protocols for managing patients at high risk of BF development.

## Conclusions

Biochemical analysis of cyst contents proves to be a highly specific and accurate method for verifying biliary fistulas (BF) in liver echinococcosis, particularly for detecting occult cases that might otherwise go unnoticed until postoperative bile leakage occurs. While conventional blood biochemical markers, including bilirubin, ALP, GGT, ALT, and AST, showed limited diagnostic value, cyst fluid analysis - specifically total and conjugated bilirubin levels - demonstrated 100% specificity and 92% accuracy. The significantly higher bilirubin levels in cysts with BF, especially in obvious cases (41±15.9 μmol/L vs. 6±3.7 μmol/L in occult BF), highlight the importance of intraoperative biochemical evaluation in improving diagnostic precision and guiding surgical decision-making.

Although this method enhances BF detection, some limitations, including moderate sensitivity (60%) and the retrospective, single-center study design, must be considered. Future research should focus on validating these findings in larger, multicenter studies and integrating biochemical analysis into standardized diagnostic protocols. By improving preoperative and intraoperative BF detection, these insights can help optimize surgical strategies and reduce postoperative complications in liver echinococcosis patients.
